# Epigenetic Modulation of IL‐7 and IL‐10: Toward Personalized Immune Therapies in Viral Epidemics

**DOI:** 10.1155/jimr/9467657

**Published:** 2026-01-08

**Authors:** Zerrin Yulugkural, Mustafa Yildiz, Ertugrul Topcu, Habibe Tülin Elmaslar Mert, Alp Temiz

**Affiliations:** ^1^ Department of Infectious Diseases and Clinical Microbiology, Trakya University, Edirne, Türkiye, trakya.edu.tr; ^2^ Department of Biophysics, Trakya University, Edirne, Türkiye, trakya.edu.tr; ^3^ Department of Medicine 3 - Rheumatology and Immunology, Friedrich-Alexander-Universität Erlangen-Nürnberg and Universitätsklinikum Erlangen, Erlangen, Germany, fau.eu; ^4^ Deutsches Zentrum für Immuntherapie (DZI), Friedrich-Alexander-Universität Erlangen-Nürnberg and Universitätsklinikum Erlangen, Erlangen, Germany, fau.eu

**Keywords:** cytokines, DNA methylation, epidemics, epigenesis, genetic, immune response

## Abstract

**Background:**

Host immune responses, including cytokine production, shape the severity of viral epidemics. Epigenetic mechanisms such as DNA methylation regulate cytokine gene expression and may contribute to immune dysregulation in severe disease.

**Methods:**

This study analyzed interleukin‐7 (IL‐7), IL‐8, and IL‐10 promoter methylation in 145 COVID‐19 patients (91 wards, 54 intensive care units (ICUs)), excluding 12 patients receiving epigenetically active drugs. Peripheral blood DNA underwent bisulfite conversion, followed by PCR and gel electrophoresis. Gene‐specific methylation levels were quantified using beta values.

**Results:**

IL‐7 was significantly hypermethylated overall (β = 0.835, *p* < 0.001), especially in ICU patients (β = 0.863, *p* = 0.001), independent of age, mortality, and malignancy. An interaction between age and ICU status indicated group‐specific effects. IL‐10 showed significant hypomethylation (β = 0.243, *p* < 0.001), while IL‐8 methylation did not differ significantly (*p* = 0.373). ICU patients had higher mortality (26% vs 5.5%, *p* < 0.001).

**Conclusion:**

IL‐7 hypermethylation may impair T‐cell–mediated immunity in severe cases, while IL‐10 hypomethylation may reflect enhanced immunosuppression. These findings suggest a role for epigenetic cytokine regulation in disease progression and may guide future immunomodulatory strategies.

## 1. Introduction

History records not only the developments, achievements, wars, and their consequences driven primarily by human actions, but also the stories of epidemics caused by microorganisms that share the world as a habitat, significantly impact societies, and lead to serious consequences. The COVID‐19 disease, which most recently emerged at the end of 2019, has spread rapidly all over the world and has turned into a pandemic, and is still continuing its effects [1]. COVID‐19 can progress to acute respiratory distress syndrome (ARDS) and has led to significant mortality, particularly in the initial phases of the pandemic [2]. Disease severity depends on factors such as viral load, age, sex, and underlying health status, as well as the immune responses of the host [3, 4]. The virus triggers various immune responses by interacting with the cellular and molecular components of the immune system. Among these, the overproduction of proinflammatory cytokines and the activation of immune cells are critical [5].

CD4+ and CD8+ T‐cells are crucial in mounting virus‐specific immune responses. Their activation leads to antiviral effects, including controlling viral replication, regulating inflammation, and clearing infected cells. However, an overactive immune response, characterized by excessive cytokine production, can result in tissue damage. In severe COVID‐19 cases, this excessive response, often termed a “cytokine storm,” can lead to life‐threatening conditions such as ARDS [6].

Epigenetic mechanisms, particularly DNA methylation, may also be involved in the body’s response to SARS‐CoV‐2 infection. DNA methylation is a heritable modification where methyl groups are added to cytosine bases in the DNA sequence. This alteration can regulate gene expression, affecting cellular functions [7, 8]. Understanding the molecular and immune mechanisms in COVID‐19 may provide insights into the disease’s progression and help identify at‐risk groups. This knowledge is crucial for public health planning and resource allocation during pandemics.

Mechanisms such as the activation of CD4+ and CD8+ T‐cells and the production of proinflammatory cytokines allow the immune system, both innate and acquired, to mount an antiviral response. These responses are essential for controlling viral replication, curbing the spread of the virus, and clearing infected cells [9]; [10]. However, in some cases, tissue damage from the virus can lead to the overproduction of cytokines, which in turn trigger the activation of macrophages and granulocytes—key players in inflammation [1, 11]. In SARS‐CoV‐2 infection, this exaggerated immune response, referred to as a cytokine storm, has been observed in clinical and imaging findings, particularly in severe COVID‐19 cases that lead to ARDS [1].

Patients with varying degrees of COVID‐19 severity exhibit differences in their chemokine and cytokine profiles. Elevated plasma levels of interferon‐gamma (IFN‐γ), tumor necrosis factor‐alpha (TNF‐α), granulocyte colony‐stimulating factor (G‐CSF), monocyte chemoattractant protein‐1 (MCP‐1), macrophage inflammatory proteins (MIP)‐1A and MIP‐1B, and interleukins such as IL‐1β, IL‐7, IL‐8, and IL‐10 have been detected in SARS‐CoV‐2 positive individuals [11].

Interleukin‐7 (IL‐7) is crucial for T‐cell survival and proliferation, playing a significant role in immune regulation during viral infections [12, 13]. Clinical trials have investigated IL‐7 as a potential therapy to restore T‐cell counts and enhance immune function in COVID‐19 patients [14]. IL‐8, responsible for recruiting neutrophils to the lungs to fight the virus, can also contribute to lung inflammation when overproduced, leading to ARDS [15, 16]. Meanwhile, IL‐10 helps prevent immune‐mediated tissue damage by suppressing proinflammatory cytokines. However, excessive IL‐10 can weaken the immune response, allowing the virus to persist and possibly contributing to long COVID [17].

Epigenetic changes, such as DNA methylation, influence gene expression without altering the genetic code and may be inherited [18]. These modifications play a role in several biological processes, including cell differentiation, aging, and disease [19]. DNA methylation typically occurs at CpG islands and is associated with gene silencing or reduced expression [20].

Epigenetic mechanisms, including DNA methylation, can influence cytokine production by modulating gene expression. DNA methylation patterns in cytokine gene promoters directly affect transcriptional activity, with hypermethylation often leading to reduced gene expression and hypomethylation promoting increased expression. Maintaining an appropriate balance of these processes is critical for proper immune function [21].

Investigating the epigenetic and immune responses in COVID‐19 patients through molecular methods can provide valuable insights. Analyzing gene regions that encode specific cytokines and observing CpG islands in these regions can help determine the genetic coding that regulates cytokine production [22]. This analysis could allow for the creation of detailed profiles of cytokine‐related gene regions in patients with varying degrees of COVID‐19 severity, helping to predict clinical outcomes [23]. Additionally, reassessing antiviral treatment duration and determining patient eligibility for suppressive therapy—either to counteract excessive immune responses or to address immunosuppression—may guide treatment strategies. Understanding these molecular mechanisms is crucial for effective disease management. Epigenetic regulation is not limited to DNA methylation but also includes histone modifications, noncoding RNAs, and chromatin remodeling, all of which can shape immune responses [24]. In the present study, we focused specifically on DNA methylation because it represents a stable and widely measurable modification with a well‐defined role in cytokine gene regulation at CpG sites [25]. Nevertheless, additional epigenetic mechanisms are likely to contribute to immune dysregulation in COVID‐19 and should be investigated in future studies.

## 2. Materials and Methods

### 2.1. Clinical Samples and Data Collection

A total of 157 COVID‐19 patients were included in this study. Age, sex, and comorbidities such as hypertension, renal failure, malignancy, immunosuppressive therapy, and coronary artery disease were reviewed and obtained from the patients’ medical records.

To ensure valid IL‐7, IL‐8, and IL‐10 epigenetic analyses, patients on medications with known epigenetic effects were excluded. This applied to 12 out of 157 patients. These drugs, including rituximab, methotrexate, cyclophosphamide, venetoclax, bortezomib, lenalidomide, platinum‐based chemotherapeutic agents like cisplatin, carboplatin, and oxaliplatin, paclitaxel, and trastuzumab, significantly alter DNA methylation, histone modifications, and cytokine gene expression. Rituximab affects cytokine expression through IL‐10 inhibition and B‐cell epigenetic reprograming [26, 27]. Methotrexate modifies DNA methylation and suppresses inflammatory cytokines [28]. Cyclophosphamide shifts immune responses by altering cytokine expression [29]. Venetoclax and platinum‐based agents induce genome‐wide methylation changes contributing to drug resistance [30, 31]. Lenalidomide modulates histone modifications [32], while trastuzumab influences immune responses [33]. Given these substantial epigenetic effects, exclusion of these medications was necessary to minimize confounding and ensure reliable study results.

Blood samples for DNA analysis were taken during routine laboratory tests in the intensive care unit (ICU) and the COVID‐19 wards managed by the University Faculty of Medicine, Department of Infectious Diseases, without the need for additional invasive procedures. None of the patients were vaccinated.

Patients were admitted to the ICU based on the criteria applied by Trakya University Faculty of Medicine Hospital. These criteria included a respiratory rate of ≥30 breaths per minute, signs of dyspnea and respiratory distress, oxygen saturation below 90% despite receiving ≥5 L/min nasal oxygen support, or a partial oxygen pressure (PaO2) below 70 mmHg under the same conditions. Additional criteria encompassed a PaO2/FiO2 ratio <300, lactate levels >4 mmol/L, bilateral infiltrations or multilobar involvement on chest imaging, hypotension (systolic blood pressure <90 mmHg, a reduction of >40 mmHg from baseline, or mean arterial pressure <65 mmHg), and signs of impaired tissue perfusion. Furthermore, patients with organ dysfunction (such as abnormal renal or liver function tests, thrombocytopenia, or confusion), immunosuppressive conditions, uncontrolled multiple comorbidities, elevated troponin levels, or arrhythmias were also considered for ICU admission. These criteria were implemented as part of routine clinical practice to ensure standardized admission decisions.

This study was approved by the Republic of Turkey Ministry of Health’s COVID‐19 Scientific Research Platform (Form Name: ‐2020‐12‐18T11_54_07) and received approval from the University Faculty of Medicine Scientific Research Ethics Committee (TUTF‐BAEK) under Protocol Code TUTF‐BAEK 2020/440, Decision No. 20/06, dated December 7, 2020. All procedures were carried out in accordance with the principles outlined in the Declaration of Helsinki, Good Clinical Practice Guidelines, and TUTF‐BAEK Guidelines. Written informed consent was obtained from all patients or their legal proxies prior to their participation in the study.

### 2.2. Total DNA Isolation

DNA was purified from peripheral blood samples using the Thermo Fisher Purelink Genomic DNA Mini Kit following the manufacturer’s protocol. No cell sorting was performed prior to DNA isolation, and therefore, the methylation measurements represent composite signals from multiple immune cell populations. Briefly, 200 µL of blood lysate was mixed with 20 µL Proteinase K and 200 µL Lysis Buffer, vortexed, and incubated at 56°C for 10 min. After adding 200 µL ethanol, the lysate was transferred to spin columns and centrifuged. Washing steps were performed with Wash Buffer I and II, and DNA was eluted with Elution Buffer. DNA quantity and purity were assessed using the NanoDrop 2000 c spectrophotometer. Samples with A260/A280 ratios between 1.8 and 2.0 and A260/A230 ratios greater than 1.5 were considered acceptable for downstream bisulfite conversion and PCR analysis.

### 2.3. Bisulfite Conversion Protocol

Bisulfite conversion was performed using the Thermo Scientific EpiJET Bisulfite Conversion Kit. DNA samples (200 ng) were adjusted to 20 µL with water, then 120 µL of Modification Reagent was added. Samples were mixed, centrifuged, and placed in a thermal cycler. Protocol A (98°C for 10 min and 60°C for 150 min) was used for denaturation and conversion. The converted DNA was purified using Binding, Wash, and Desulfonation Buffers, then eluted with Elution Buffer. DNA was quantified using UV absorbance measurements and stored at −20°C for further use.

### 2.4. PCR Amplification

Amplification of bisulfite‐converted DNA was conducted using AmpliTaq Gold 360 Master Mix. PCR reactions were prepared with 2 µL of eluted DNA and primers specific for methylated (M) and unmethylated (U) sequences, where F denotes the forward primer, and R denotes the reverse primer, as follows: IL‐7MF: TGAGTAGGTGTATGTATAGTAGACGG and R: CAAACTAAATTATTAAAAACGAACGA. IL‐7 U F: GAGTAGGTGTATGTATAGTAGATGG and R: CAAACTAAATTATTAAAAACAAACAA. IL‐8MF: AAAATTTTCGTTATATTTCG and R: TCCGATAACTTTTTATATCAT. IL‐8 U F: AAATTTTTGTTATATTTTG and R: TCCAATAACTTTTTATATCAT. IL‐10MF: TTTATAGTTGAGGGTTTTTGCG and R: TTTATAGTTGAGGGTTTTTGCG. IL‐10 U F: TGAATGAGAATTTATAGTTGAGG and R: TTTCAATTTTTACATCATAAAC. The PCR conditions included an initial denaturation at 95°C for 5 min, followed by 10 cycles of 95°C for 1 min, 58°C for 1.5 min, and 72°C for 1 min, and then 30 cycles of 95°C for 1 min, 58°C for 1 min, and 72°C for 1 min, with a final extension at 72°C for 5 min.

### 2.5. Gel Electrophoresis

PCR products were analyzed using agarose gel electrophoresis. A 2% agarose gel was prepared with TBE buffer and stained with Safeview Classic dye. DNA samples were mixed with Orange G loading dye, loaded onto the gel, and electrophoresed. Gels were visualized using a UV transilluminator, and band intensities were analyzed with GelAnalyzer 19.1 software to calculate methylation percentages (Supporting Information: Figure S1).

### 2.6. Statistical Analysis

Methylation levels were assessed and compared as beta values. The methylation beta value was calculated as *β* = M/(M + U + 100), where M represents methylated band densities, U represents unmethylated band densities, and 100 is a constant. We filtered out samples where the combined methylated and unmethylated (M + U) band densities were less than 100. This step was necessary because low M + U band densities can introduce significant noise and skew logarithmic calculations. By excluding these samples, we ensured that minor fluctuations did not cause large variances, leading to more accurate results. To address the challenges of indeterminate forms caused by zero values in band densities, Laplace smoothing was applied. This approach enabled the calculation of meaningful values within the [0,1] range, enhancing the robustness of the results and the credibility of the study.

Given the bounded nature of methylation beta values and their often skewed or bimodal distribution, assumptions of normality and homoscedasticity required for parametric tests were not met; therefore, non‐parametric statistical methods were employed. Specifically, the Wilcoxon one‐sample median test was employed to assess whether the methylation levels for each IL group differed significantly from 0.5, which represents a baseline indicating no hyper‐ or hypomethylation. Additionally, the Wilcoxon rank‐sum test was used to compare methylation levels between the ICU and ward groups for each IL. To control for effect size, the rank‐biserial correlation (r_rb_) was calculated, providing a robust evaluation of the strength and direction of the relationship between the groups. Analyses were conducted using the R statistical language (version 4.4.1; R Core Team, 2024) on Windows 10 x64 (build 19,045), using the packages ggrain, janitor, lubridate, Hmisc, ggpubr, report, tibble, FSA, ggstatsplot, table1, dlookr, gtsummary, cardx, ggplot2, forcats, stringr, tidyverse, readxl, dplyr, purrr, readr, tidyr, colorspace, and kableExtra. Statistical significance was accepted at *p* < 0.05.

## 3. Results

### 3.1. Patient Characteristics

**Figure 1:**
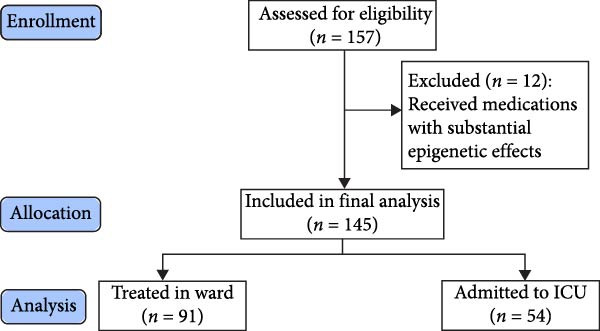
CONSORT flow diagram. Of 157 patients assessed, 12 were excluded. The final cohort (*n* = 145) included 91 ward‐treated and 54 ICU‐admitted patients.

A total of 157 patients were initially evaluated, of which 12 patients were excluded due to receiving medications with substantial epigenetic effects, leaving 145 patients included in the final analysis. Among these, 91 patients were treated in the ward, and 54 required ICU admission based on clinical criteria (Figure 1). The mean age of ICU patients was significantly higher at 70 (SD = 16) years compared to 58 (SD = 14) years for ward patients (*p* < 0.001). Sex distribution was comparable between groups, with females comprising 53% in the ward group and 50% in the ICU group (*p* = 0.7). Hypertension was significantly more prevalent among ICU patients (63% vs 43% in ward; *p* = 0.019). Other comorbidities (renal insufficiency, coronary artery disease, and immunosuppressive therapy) were numerically higher in ICU patients but not statistically significant. Malignancy was significantly more common in ICU patients (20% vs 7.7% in ward; *p* = 0.025). Finally, the ICU group experienced a markedly higher mortality rate (26% vs 5.5% in ward; *p* < 0.001) (Table 1).

**Table 1:** Baseline characteristics of patients by ICU admission status.

Characteristic	Ward, *N* = 91^1^	ICU, *N* = 54^1^	*p*‐Value^2^
Age	58 (14)	70 (16)	<0.001
Sex	—	—	0.7
Female	48 (53%)	27 (50%)	—
Male	43 (47%)	27 (50%)	—
Hypertension	39 (43%)	34 (63%)	0.019
Diabetes mellitus	29 (32%)	12 (22%)	0.2
Renal insufficiency	6 (6.7%)	8 (15%)	0.11
Coronary artery disease	17 (19%)	15 (28%)	0.2
Immunosuppressive therapy	10 (11%)	7 (13%)	0.7
Malignancy	7 (7.7%)	11 (20%)	0.025
Mortality	5 (5.5%)	14 (26%)	<0.001

^1^Mean (SD); *n* (%).

^2^Wilcoxon rank sum test; Pearson’s Chi‐squared test.

### 3.2. Cytokine Methylation Overview

Methylation levels of IL‐7, IL‐8, and IL‐10 were evaluated using the Wilcoxon signed‐rank test, as the Shapiro–Wilk test confirmed non‐normal distributions (*p* < 0.001 for each). IL‐7 displayed significant hypermethylation, with a median methylation level of 0.835 (*n* = 126; *p* < 0.001) and a very large positive effect size (rrb = 0.99, 95% CI: 0.99–1.00) (Figure 2A). IL‐8 methylation showed no significant deviation from 0.5 (median = 0.538, *p* = 0.986; rrb = –0.002, 95% CI: –0.22 to 0.216) (Figure 2C). Conversely, IL‐10 exhibited significant hypomethylation, with a median of 0.243 (*p* < 0.001) and a large negative effect size (rrb = –0.736, 95% CI: –0.83 to –0.60) (Figure 2E).

Figure 2Comparison of beta values for IL‐7, IL‐8, and IL‐10 between ward and ICU patients. Panels (A), (C), and (E) show histograms of beta values for IL‐7, IL‐8, and IL‐10, respectively. The red dashed lines represent the median values, and the blue dashed lines indicate a reference threshold. Panels (B), (D), and (F) display violin plots comparing beta values between patients in the ward and ICU groups for IL‐7, IL‐8, and IL‐10, respectively. Statistical results from the Wilcoxon rank‐sum test (W) and the rank‐biserial correlation (r) are provided in each plot. The red horizontal dashed lines in the violin plots indicate the overall median beta value for each cytokine.

**Figure:**
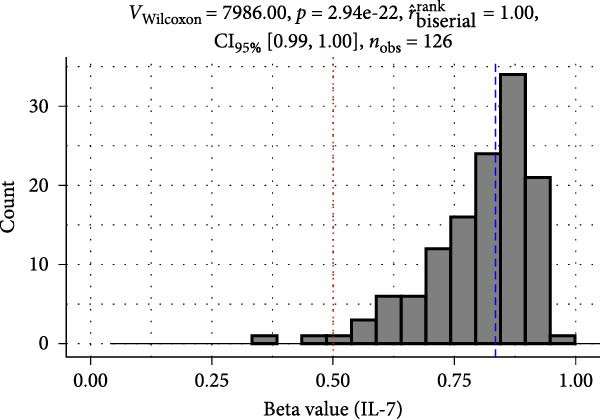
(A)

**Figure:**
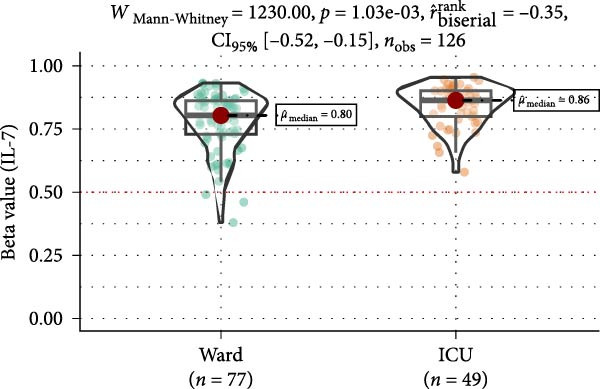
(B)

**Figure:**
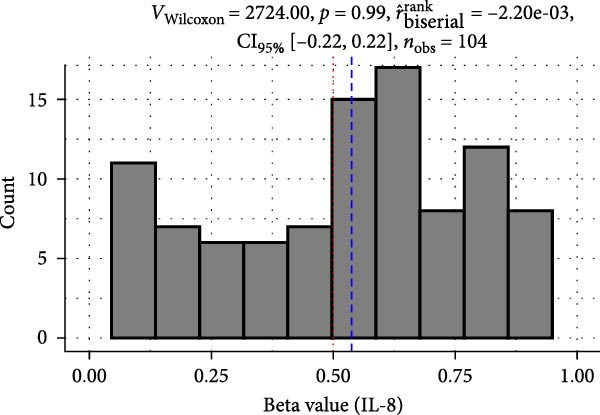
(C)

**Figure:**
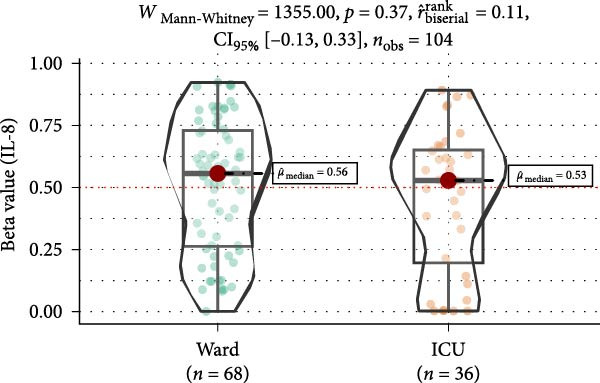
(D)

**Figure:**
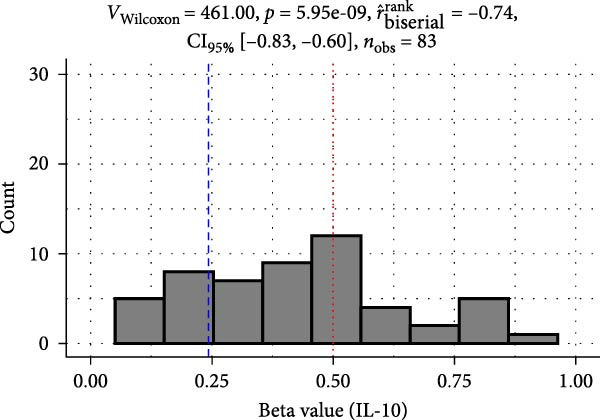
(E)

**Figure:**
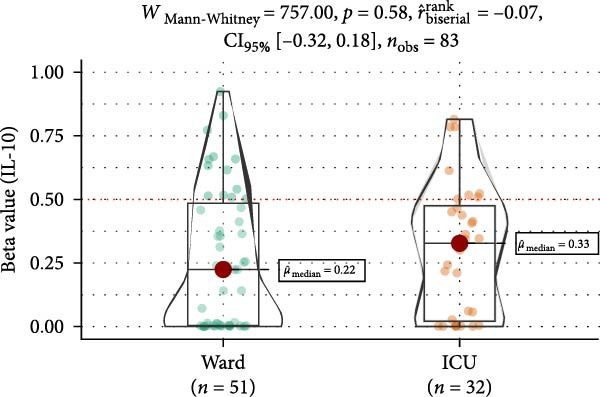
(F)

### 3.3. Comparisons by Clinical Group

Comparing methylation between ward and ICU patients revealed a significant difference in IL‐7. The ICU group showed higher median IL‐7 methylation (0.863) versus the ward group (0.835; *p* = 0.001) with a moderate negative effect size (rrb = –0.348, 95% CI: –0.516 to –0.154) (Figure 2B). In contrast, IL‐8 methylation did not differ significantly between ward (median = 0.556) and ICU patients (0.528; *p* = 0.373) (Figure 2D), and IL‐10 methylation showed no difference between ward (median = 0.224) and ICU patients (0.327; *p* = 0.37) (Figure 2F).

### 3.4. Age, Mortality, Malignancy, and IL‐7 Methylation

To assess whether the observed IL‐7 methylation difference between ICU and ward patients could be attributed to age, mortality, or malignancy, a series of linear regression models was constructed.

Two linear regression models were used to explore the association between age and IL‐7 methylation. The first model included ICU status and age as independent, additive predictors, assuming that the effect of age on methylation does not depend on ICU status. In this model, ICU patients tended to have higher IL‐7 methylation than ward patients (*β* = 0.051, *p* = 0.018), while age was not a significant predictor (*p* = 0.21). The second model included an interaction term between ICU status and age to test whether the effect of age on methylation depends on the clinical setting. This interaction was statistically significant (*p* = 0.042), indicating that the effect of age differed by group: IL‐7 methylation increased with age in ward patients but decreased with age in ICU patients. This crossover interaction suggests that the relationship between age and IL‐7 methylation is modified by clinical severity.

In a separate model evaluating the impact of mortality, ICU status again remained significantly associated with IL‐7 hypermethylation (*p* < 0.001). Mortality was not a significant predictor (*p* = 0.29), and no significant interaction was observed between ICU status and mortality (*p* = 0.67). These findings indicate that mortality does not account for the ICU‐associated methylation difference.

Finally, malignancy—which was more prevalent in ICU patients—was also considered. ICU status remained a significant predictor of IL‐7 hypermethylation after adjusting for malignancy (*β* = 0.063, *p* = 0.002), while malignancy itself showed no significant association (*p* = 0.98). An additional model including an interaction term between ICU status and malignancy showed a nonsignificant trend (*p* = 0.093), but malignancy alone remained non‐significant (*p* = 0.20).

Taken together, these models consistently show that the elevated IL‐7 methylation observed in ICU patients is not explained by age, mortality, or malignancy. Instead, it appears to reflect disease severity‐related epigenetic modulation specific to critical illness.

## 4. Discussion

Our study’s descriptive statistics offer valuable insights into patient characteristics. ICU patients were significantly older than those treated in the ward, with a mean age of 70 years compared to 58 years (*p* < 0.001). This age difference underscores the higher vulnerability of older patients to severe COVID‐19, aligning with existing literature that identifies age as a critical risk factor for severe outcomes [34]. Additionally, while the distribution of sex was comparable between the ward and ICU groups, the presence of certain comorbidities, particularly hypertension and malignancy, was significantly higher among ICU patients. This finding emphasizes the role of comorbid conditions in exacerbating the severity of COVID‐19 and highlights the need for close monitoring of such patients. Furthermore, the stark difference in mortality rates between the ICU and ward groups (26% vs 5.5%) underscores the critical impact of disease severity on patient outcomes. These descriptive statistics provide a foundational context for understanding the subsequent findings on cytokine methylation and their potential implications for disease progression and patient management.

Hypermethylation of IL‐7 was observed in our patients, and those requiring ICU care showed particularly high methylation levels. IL‐7 hypermethylation leads to gene silencing and reduces cytokine production. Reduced IL‐7 production may worsen patient outcomes, possibly necessitating ICU admission. IL‐7 is under investigation as an immunotherapy for infectious diseases. Clinical trials are exploring its potential to restore T‐cell counts, improve immune function in COVID‐19 patients, and enhance viral defense [14]. Some reports have shown that serum IL‐7 levels are significantly higher in severe COVID‐19 cases compared to moderate ones [35] and that IL‐7 levels in ICU patients are significantly elevated [1].

Although IL‐7 methylation levels and patient age both differed significantly between ICU and ward groups, our regression analyses demonstrate that age does not account for the observed methylation difference. ICU status remained a significant predictor of IL‐7 hypermethylation even after adjusting for age, and age alone was not independently associated with methylation levels. Moreover, a significant interaction between ICU status and age revealed a group‐specific pattern: while older age was linked to higher IL‐7 methylation in ward patients, the opposite trend was seen in ICU patients. This crossover effect suggests that age influences IL‐7 methylation differently depending on clinical severity, supporting the idea of distinct epigenetic responses in critically ill individuals.

Similarly, neither mortality nor malignancy—despite being more frequent in ICU patients—explained the methylation differences. ICU status retained its strong association with IL‐7 hypermethylation after accounting for each variable, and no significant interactions were found. Collectively, these findings indicate that the increased IL‐7 methylation in ICU patients is not confounded by age, mortality, or malignancy, and instead likely reflects epigenetic modulation linked directly to the biological processes underlying severe COVID‐19. The difference in IL‐7 methylation between ICU and ward groups was not mediated by mortality, as death was not a significant predictor and showed no interaction with ICU status. This suggests that the ICU‐related increase in methylation is independent of survival outcome.

In contrast to IL‐7, our analysis revealed no significant difference in IL‐8 methylation levels, with a median value of 0.54, indicating a balanced methylation status. This suggests that IL‐8 expression may not be directly influenced by methylation changes in the context of COVID‐19. Clinically, IL‐8 is recognized for its role in recruiting neutrophils to infection sites, which is crucial in the early immune response but can also contribute to excessive inflammation and lung damage in severe cases. Despite the balanced methylation status, serum IL‐8 levels have been reported to be elevated in severe cases, potentially due to regulatory mechanisms independent of methylation. For instance, IL‐8 levels were higher in non‐severe cases compared to severe cases [36]. Additionally, several inflammatory cytokines were elevated in severe cases compared to nonsevere cases, with IL‐8 and IL‐10 showing notable differences [11]. However, serum IL‐8 concentrations in severe cases were lower than in moderate cases, though the difference was not statistically significant [35]. The absence of significant methylation differences between ICU and ward patients further supports the notion that IL‐8’s contribution to disease severity is likely due to post‐transcriptional regulation or other immune modulators rather than epigenetic changes.

Our study demonstrated significant hypomethylation of IL‐10, with a median methylation level of 0.24, particularly among ICU patients. Hypomethylation is typically associated with increased gene expression, which aligns with the elevated IL‐10 serum levels observed in severe COVID‐19 cases. For instance, serum concentrations of IL‐10 were reported to be higher in non‐severe cases compared to severe cases [36]. IL‐10 is an anti‐inflammatory cytokine that plays a dual role in modulating immune responses by limiting excessive inflammation to prevent tissue damage. However, in the context of COVID‐19, increased IL‐10 levels due to hypomethylation may contribute to an overly immunosuppressive environment, potentially allowing the virus to persist and leading to worse outcomes in critically ill patients. This immunosuppressive state may also help explain the persistence of symptoms observed in “long COVID” patients, where chronic inflammation and immune dysregulation are common features [17].

Beyond DNA methylation, other epigenetic mechanisms such as histone modifications, noncoding RNAs, and chromatin remodeling play important roles in immune regulation and disease outcomes. Our study did not evaluate these additional processes, but they may be highly relevant, particularly in the context of long COVID, where persistent immune dysregulation has been described. Integrating multiple layers of epigenetic information in future research will provide a more comprehensive understanding of immune modulation during viral infections [24].

Cytokines do not act in isolation but in complex networks that coordinate immune responses [37]. The functional effects of IL‐7, IL‐8, and IL‐10, therefore, need to be interpreted within the broader cytokine milieu and in conjunction with acute‐phase reactants and other modulators of inflammation. Our focus on three ILs was hypothesis‐driven, but we acknowledge that the outcome of viral infections such as COVID‐19 is shaped by the interplay of multiple immune mediators. Future studies should therefore analyze cytokine methylation and expression patterns in combination to better capture the integrated nature of immune regulation.

This study has several limitations. The sample size, while sufficient for statistical analysis, may not fully capture the variability in DNA methylation patterns across diverse populations, and larger multicenter studies are required for validation. DNA methylation was assessed in peripheral blood, which may not reflect tissue‐specific patterns in the lung or other immune compartments, and because DNA was extracted from whole blood without cell sorting, differences in leukocyte composition could have influenced the results. The analysis was restricted to IL‐7, IL‐8, and IL‐10; other cytokines, such as IFN‐γ, which is strongly regulated by promoter methylation, were not assessed. Additional epigenetic processes, including histone modifications, noncoding RNAs, and chromatin remodeling, were also beyond the scope of this work but may play an important role, particularly in long COVID. Parallel gene expression and protein‐level measurements were not available, limiting conclusions about functional consequences; integrated methylation–transcriptome–proteome studies would provide stronger causal inference. Factors such as genetic background, prior infections, and environmental influences were not measured, which could further affect methylation status. Finally, although patients receiving medications with known epigenetic effects were excluded, other unmeasured exposures may still contribute to variability. Future research should incorporate longitudinal sampling, functional assays, and multi‐layer epigenetic profiling to better define the role of these mechanisms in immune responses during viral epidemics.

In conclusion, this study demonstrates significant differences in cytokine methylation profiles between patients with varying disease severity during viral epidemics. IL‐7 hypermethylation, particularly in ICU patients, suggests reduced immune function, while IL‐10 hypomethylation may contribute to excessive immune suppression. These epigenetic modifications may play a crucial role in disease progression, influencing immune regulation and patient outcomes. Given the increased prevalence of older age, hypertension, and malignancy among ICU patients, targeted approaches integrating both epigenetic and clinical risk factors could improve disease management. Future research should explore the functional impact of these modifications and assess their potential as biomarkers for risk stratification and personalized treatment strategies in viral epidemic settings.

## Ethics Statement

This study was approved by the Republic of Turkey Ministry of Health’s COVID‐19 Scientific Research Platform (Form Name: ‐2020‐12‐18T11_54_07) and received approval from the University Faculty of Medicine Scientific Research Ethics Committee (TUTF‐BAEK) under Protocol Code TUTF‐BAEK 2020/440, Decision No. 20/06, dated December 7, 2020. All procedures were carried out in accordance with the principles outlined in the Declaration of Helsinki, Good Clinical Practice Guidelines, and TUTF‐BAEK Guidelines. Written informed consent was obtained from all patients or their legal proxies prior to their participation in the study.

## Disclosure

No patients were involved in the design of this study.

## Conflicts of Interest

The authors declare no conflicts of interest.

## Author Contributions

Study design: Zerrin Yulugkural and Alp Temiz. Data collection: all authors. Laboratory: Mustafa Yildiz and Alp Temiz. Writing of the manuscript: all authors. Statistical analysis: Alp Temiz. Tables and figures: Alp Temiz. Supervision, resources, and funding: Zerrin Yulugkural and Alp Temiz.

## Funding

This study was supported by the Trakya University Scientific Research Projects Unit ‐ Turkey (Grant Number: 2022/106). Open Access funding enabled and organized by Projekt DEAL.

## Supporting Information

Additional supporting information can be found online in the Supporting Information section.

## Supporting information


**Supporting Information** Figure S1. Representative agarose gel electrophoresis image of methylation‐specific PCR products. PCR products were separated on a 2% agarose gel in TBE buffer, stained with Safeview™ Classic dye, and visualized under UV light. Band intensities were quantified using GelAnalyzer 19.1 to calculate methylation percentages.

## Data Availability

The data that support the findings of this study are available from the corresponding author upon reasonable request.
