# Supplementary figures and images for: Epigenetic Modulation of IL‐7 and IL‐10: Toward Personalized Immune Therapies in Viral Epidemics
Source: J Immunol Res. 2026 Jan 8;2026:9467657. doi: 10.1155/jimr/9467657 (PMC12780970; doi:10.1155/jimr/9467657)

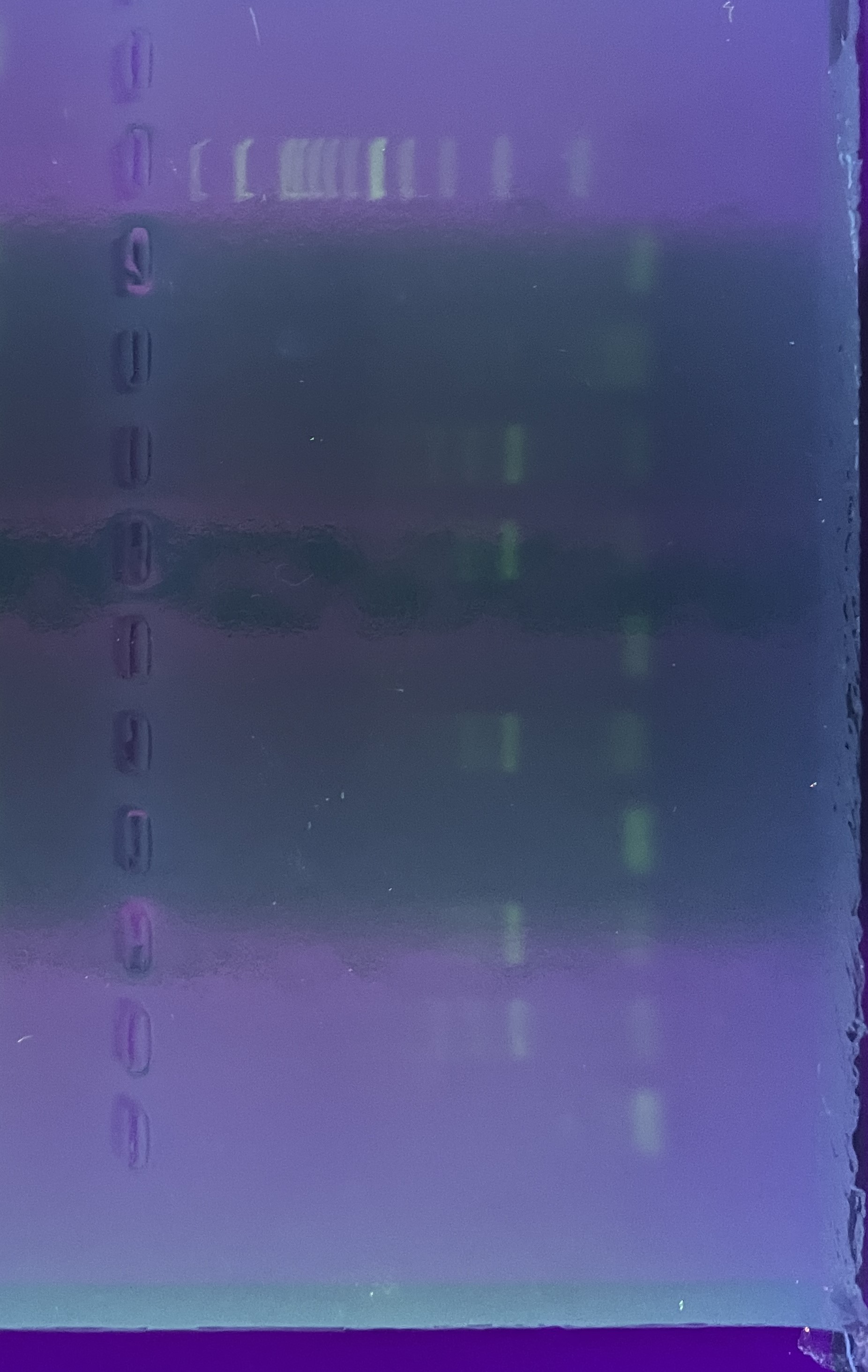

Supplement: Supplementary file 1 — Supporting Information Figure S1. Representative agarose gel electrophoresis image of methylation‐specific PCR products. PCR products were separated on a 2% agarose gel in TBE buffer, stained with Safeview™ Classic dye, and visualized under UV light. Band intensities were quantified using GelAnalyzer 19.1 to calculate methylation percentages. [file JIMR-2026-9467657-s001.jpg]
